# Clonidine Inhibits Interictal-like Epileptiform Events in Prefrontal Cortex Pyramidal Neurons

**DOI:** 10.3390/ijms27062722

**Published:** 2026-03-17

**Authors:** Weronika Kołba, Dominika Herbst, Bartłomiej Szulczyk

**Affiliations:** Chair and Department of Pharmacotherapy and Pharmaceutical Care, The Medical University of Warsaw, Banacha 1B, 02-097 Warsaw, Poland

**Keywords:** clonidine, interictal-like epileptiform events, patch-clamp, ADHD, prefrontal cortex, sodium channels

## Abstract

The mechanism of action of drugs used to treat ADHD has not been fully elucidated. The aim of the study was to assess the effect of clonidine, a drug used to treat ADHD, on interictal-like epileptiform events in prefrontal cortex pyramidal neurons. Epileptiform events (lasting less than 3 s) were recorded in a zero-magnesium and elevated-potassium proepileptic extracellular solution using the patch-clamp methodology. Clonidine 100 µM reduced the frequency of epileptiform events. Moreover, clonidine hyperpolarized the membrane potential recorded in the proepileptic extracellular solution. In the constant presence of the alpha-2 adrenergic receptor antagonist idazoxan 20 µM in all solutions, clonidine 100 µM also inhibited the frequency of interictal-like epileptiform events. This suggests that clonidine inhibited the frequency of interictal events via a direct influence on ionic channels. Furthermore, clonidine inhibited tonic NMDA receptor currents and did not influence tonic AMPA currents. The tested drug inhibited fast-inactivating voltage-gated sodium currents. Blockade of NMDA currents and voltage-gated sodium currents likely contributed to the inhibition of epileptiform events exerted by clonidine. The potential translational relevance of the study is discussed.

## 1. Introduction

ADHD (attention deficit hyperactivity disorder) occurs in about 5% of children and 2.5% of adults worldwide [1]. The prevalence of this disorder has increased in recent years [2]. Working memory impairment and cognitive deterioration are commonly described in ADHD [1,3]. ADHD often coexists with epilepsy, suggesting that the pathogenesis of these disorders may involve similar mechanisms [1,4,5].

There are two types of epileptiform discharges: those occurring during epileptic seizures (long-lasting ictal epileptic events) and those occurring between seizures (short-lasting interictal epileptic discharges (IEDs)) [6,7]. There are also subclinical epileptiform discharges that occur in patients without seizures [1]. It has been found that subclinical and interictal epileptiform discharges occur more often in children with ADHD and contribute to cognitive deterioration [1].

The prefrontal cortex is involved in the pathogenesis of ADHD because this brain region plays an important role in attention [8]. The prefrontal cortex is also involved in working memory formation and, consequently, in cognitive functions that are impaired in ADHD. It has been found that clonidine, an important drug used to treat ADHD, improves working memory and cognitive functions by stimulating alpha-2 adrenergic receptors in the prefrontal cortex [3]. It may be speculated, however, that stimulating alpha-2 adrenergic receptors is not the only mechanism of action of clonidine.

In our recent publication, we found that guanfacine, an alpha-2 adrenergic receptor agonist used to treat ADHD, inhibits interictal-like epileptiform events in the prefrontal cortex [7]. We also found that voltage-gated sodium channels are involved in this effect.

The aim of the present study was to assess the influence of clonidine on interictal-like epileptiform events in the prefrontal cortex pyramidal neurons. The aim was also to elucidate the mechanism underlying this effect.

## 2. Results

**Figure 1:**
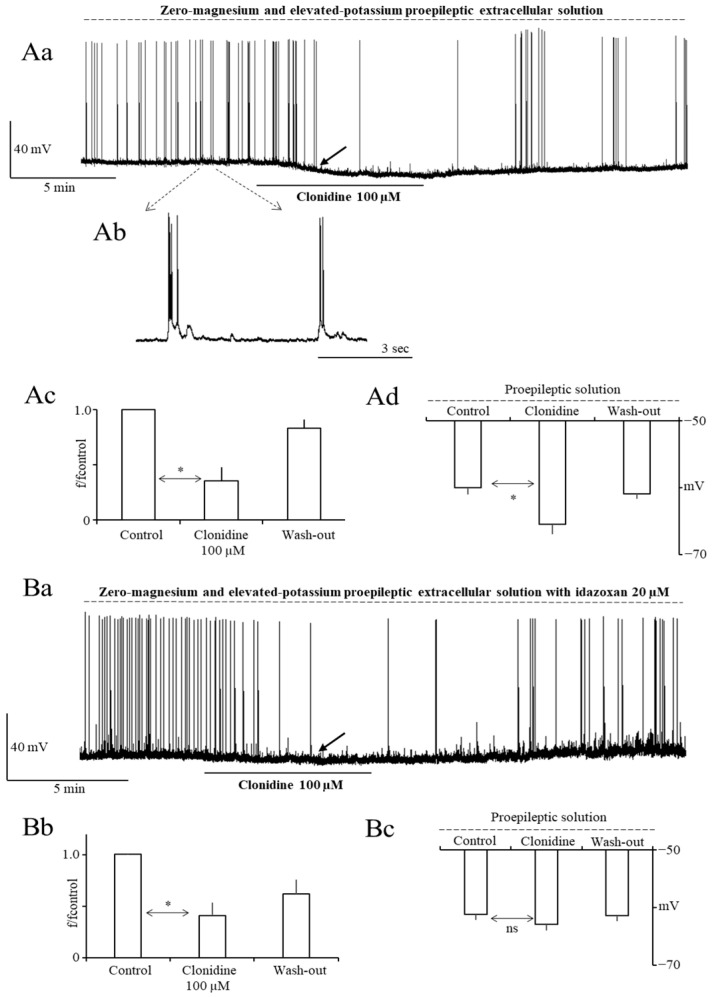
Clonidine 100 µM inhibits interictal-like epileptiform discharges (IEDs) in PFC pyramidal neurons. (**Aa**)—Example recording of IEDs in the control, after the addition of clonidine 100 µM, and after wash-out. IEDs were recorded in a proepileptic extracellular solution containing zero magnesium and elevated potassium, as indicated by a dashed line above the recording. (**Ab**)—Example of control IEDs shown on an expanded time scale. The vertical scale is the same for (**Aa**,**Ab**). (**Ac**)—Averaged normalized frequency of IEDs in the control, in the presence of clonidine 100 µM, and after wash-out. Statistical significance is indicated by an asterisk (Tukey’s post hoc test). (**Ad**)—Averaged membrane potential recorded in proepileptic extracellular solution in the control, in the presence of clonidine 100 µM, and after wash-out. Statistical significance is indicated by an asterisk (Tukey’s post hoc test). Membrane potential hyperpolarization is shown by a black solid arrow in Figure 1Aa on an example neuron. Clonidine 100 µM inhibits IEDs in the presence of the alpha-2 adrenergic receptor antagonist idazoxan 20 µM in all extracellular solutions. (**Ba**)—Example recording of IEDs in the control, after the addition of clonidine 100 µM, and after wash-out. (**Bb**)—Averaged normalized frequency of IEDs in the control, in the presence of clonidine, and after wash-out, with the statistical significance (Tukey’s post hoc test) shown by an asterisk. (**Bc**)—Averaged membrane potential recorded in the proepileptic extracellular solution with idazoxan 20 µM in the control, in the presence of clonidine 100 µM, and after wash-out.

Interictal-like epileptiform discharges (IEDs) were evoked in a proepileptic extracellular solution containing zero magnesium and elevated potassium [7]. After stabilizing the membrane potential in the physiological extracellular solution, the proepileptic extracellular solution was applied and a stable frequency of interictal epileptiform discharges (IEDs) was recorded [7]. After that, clonidine 100 µM was applied to the whole bath for 7 to 10 min. Clonidine reduced the frequency of IEDs. It was possible to obtain wash-out. The averaged control frequency of IEDs was 0.2 ± 0.04 Hz (n = 19). The averaged normalized frequency of IEDs was 1.0 ± 0.0, 0.35 ± 0.13, and 0.83 ± 0.08 in the control, in the presence of clonidine 100 µM, and after wash-out, respectively (n = 9, *p* < 0.01 (Tukey’s post hoc test control vs. clonidine), Figure 1Aa,Ac).

In the constant presence of alpha-2 adrenergic receptor antagonist idazoxan 20 µM in all solutions, clonidine 100 µM inhibited the frequency of IEDs to a similar extent as in the absence of idazoxan in the bath (see above). This indicates that alpha-2 adrenergic receptors were not involved in the inhibition of IEDs exerted by clonidine and that the blockade of IEDs occurred via direct influence on ionic channels. With idazoxan in the bath, the normalized frequency of IEDs was 1.0 ± 0.0 in the control, 0.41 ± 0.13 in the presence of clonidine 100 µM, and 0.79 ± 0.23 after wash-out (n = 10, *p* < 0.01 (Tukey’s post hoc test control vs. clonidine), Figure 1Ba,Bb).

Moreover, clonidine 100 µM hyperpolarized the membrane potential recorded in a zero-magnesium and elevated-potassium proepileptic solution, as shown by the black solid arrow in Figure 1Aa (the membrane potential was −60.1 ± 1.0 mV, −65.5 ± 1.5 mV, and −60.9 ± 0.8 mV in the control, in the presence of clonidine 100 µM, and after wash-out, respectively (Figure 1Aa,Ad, n = 9, *p* < 0.05 (Tukey’s post hoc test control vs. clonidine)). The averaged membrane potential change was 5.4 ± 1.7 mV.

In the constant presence of idazoxan 20 µM in the bath, clonidine insignificantly hyperpolarized the membrane potential recorded in the zero-magnesium and elevated-potassium proepileptic solution, as shown by the black solid arrow in Figure 1Ba (the averaged membrane potential change was 1.8 ± 0.9 mV (Figure 1Ba,Bc, n = 10, *p* > 0.05 (Tukey’s post hoc test control vs. clonidine)). The averaged membrane potential change without idazoxan in the bath was 5.4 ± 1.7 mV (*p* < 0.05; see above). Thus, alpha-2 adrenergic receptors were involved in the hyperpolarization of the membrane potential exerted by clonidine.

**Figure 2:**
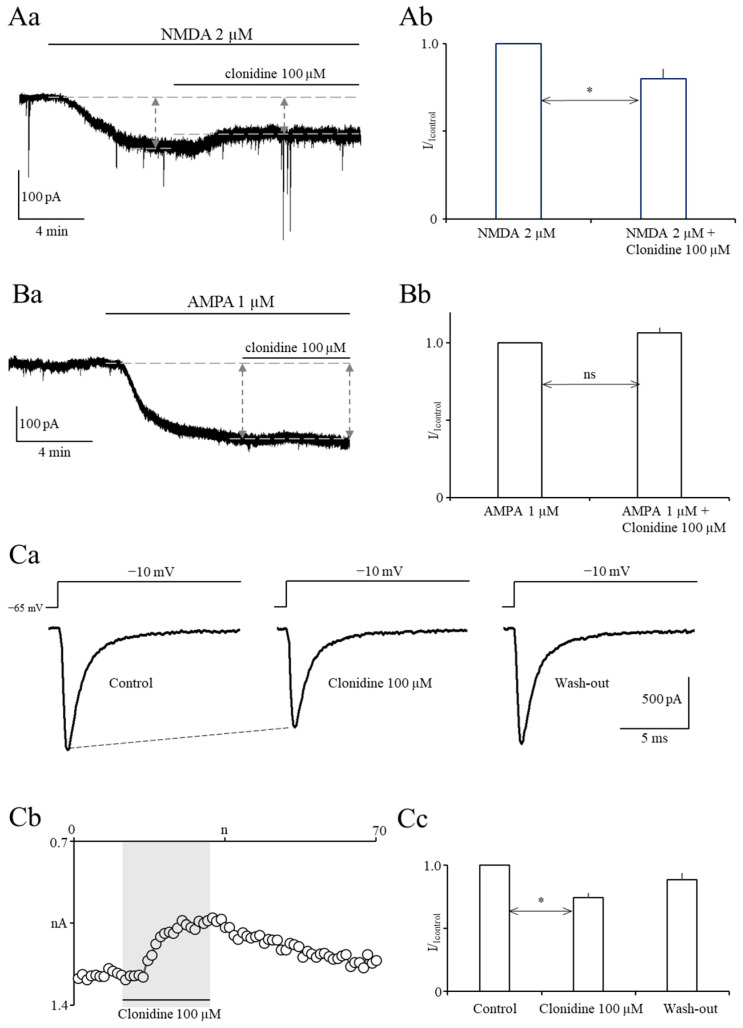
Clonidine 100 µM inhibits tonic NMDA currents and voltage-gated sodium currents but does not influence tonic AMPA currents. (**Aa**)—Original recording of NMDA current evoked by application of NMDA 2 µM to the whole bath. The upper dashed line indicates the baseline current and the lower dashed lines indicate the current after application of NMDA 2 µM. The left vertical arrow indicates the NMDA current before application of clonidine 100 µM and the right vertical arrow indicates the NMDA current after application of clonidine. (**Ab**)—Averaged normalized NMDA current before and after application of clonidine 100 µM (statistical significance is shown by an asterisk, paired *t*-test). (**Ba**)—Original recording of an AMPA current evoked by application of AMPA 1 µM to the whole bath. The upper dashed line indicates the baseline current and the lower dashed line indicates the current after application of AMPA 1 µM. The left vertical arrow indicates the AMPA current before application of clonidine 100 µM and the right vertical arrow indicates the AMPA current after application of clonidine 100 µM. (**Bb**)—Averaged normalized AMPA current before and after application of clonidine 100 µM. (**Ca**)—Example sodium current recordings in the control, after application of clonidine 100 µM, and after wash-out. Currents were evoked by rectangular voltage steps to −10 mV, as shown above the current traces. (**Cb**)—The effect of clonidine 100 µM on the sodium current is shown on an example neuron. Current traces were evoked once every ten seconds. The vertical axis shows maximal current amplitudes (white circles) in the control, in the presence of clonidine, and after wash-out. The horizontal axis shows the trace number. (**Cc**)—Averaged normalized maximal current amplitudes in the control, in the presence of clonidine 100 µM, and after wash-out (currents were normalized to control currents; statistical significance is shown by an asterisk (Tukey’s post hoc test control vs. clonidine)).

Next, we assessed the influence of clonidine 100 µM on tonic NMDA currents. NMDA 2 µM was applied for 6 to 8 min to the whole bath to evoke stable NMDA currents (left vertical arrow in Figure 2Aa). Next, NMDA 2 µM and clonidine 100 µM were coapplied for 6 to 10 min to assess the influence of clonidine on NMDA currents (right vertical arrow in Figure 2Aa). Clonidine 100 µM inhibited tonic NMDA currents (the averaged normalized NMDA current in the presence of clonidine was 0.8 ± 0.05 compared with the control NMDA current 1.0 (Figure 2Ab, n = 5, *p* < 0.05 (paired *t*-test)).

We also assessed the influence of clonidine 100 µM on tonic AMPA currents. Stable AMPA currents were evoked after application of AMPA 1 µM to the whole bath for 7 to 15 min (left vertical arrow in Figure 2Ba). After that, AMPA 1 µM and clonidine 100 µM were coapplied for 7 to 9 min to assess the effect of clonidine on AMPA currents (right vertical arrow in Figure 2Ba). Clonidine 100 µM did not influence tonic AMPA currents (averaged normalized AMPA current in the presence of clonidine was 1.06 ± 0.03 compared with the control AMPA current 1.0 (Figure 2Ba,Bb, n = 5, *p* > 0.05 (paired *t*-test)).

Finally, we assessed the influence of clonidine 100 µM on fast-activating and fast-inactivating voltage-gated sodium currents. Maximal currents were evoked by rectangular voltage steps to −10 mV (Figure 2Ca). Holding potential was −65 mV. Control currents were recorded for 2 min, and clonidine 100 µM was applied to the whole bath for 3 min. After that, wash-out was obtained (Figure 2Cb). Maximal sodium currents were inhibited by clonidine 100 µM. Averaged normalized maximal sodium currents were 1.0 ± 0.0, 0.75 ± 0.03, and 0.89 ± 0.05 in the control, after the addition of clonidine 100 µM, and after wash-out, respectively (Figure 2Ca–Cc, n = 5, *p* < 0.01 (Tukey’s post hoc test control vs. clonidine)).

## 3. Discussion

In this study, epileptiform events were recorded in a zero-magnesium and elevated-potassium proepileptic extracellular solution. These events may be regarded as interictal-like (occurring between seizures) because they lasted less than 3 s [6,7].

The absence of magnesium in the proepileptic solution unblocks NMDA receptors, whereas an increased potassium concentration depolarizes the neuronal membrane [9]. Under such conditions, the increased spontaneous release of glutamate potently stimulates facilitated NMDA receptors/channels, which generates IEDs. AMPA receptors have also been shown to be involved in the generation of IEDs [10]. This study shows that clonidine inhibits tonic NMDA currents, which may contribute to the inhibition of IEDs exerted by clonidine. In contrast, the blockade of AMPA receptors by clonidine is not involved in the inhibition of IEDs by the tested drug because we found that clonidine does not influence AMPA currents.

It has been found that presynaptic and postsynaptic voltage-gated sodium and calcium channels are involved in the generation of IEDs [11,12]. This study shows that clonidine inhibits fast-inactivating voltage-gated sodium channels, which may contribute to the inhibition of IEDs exerted by clonidine. The question arises as to which sodium channel subtypes are inhibited by clonidine. Other authors have shown that fast voltage-gated sodium currents are attributable to Nav1.1 and Nav1.2 sodium channel α-subunits in prefrontal cortex pyramidal neurons [13]. Therefore, clonidine likely inhibits one or both of these sodium channel subtypes.

Other authors suggested that clonidine antagonizes NMDA receptors in the brain stem in vivo [14]. It has also been found by other authors that clonidine inhibits Nav1.7 sodium channels in adrenal chromaffin cells [15]. To our knowledge, our findings indicate for the first time that clonidine inhibits sodium and NMDA currents in the prefrontal cortex.

Clonidine may also inhibit interictal-like epileptiform discharges by influencing other molecular effectors. It has been described by others that potassium channel openers terminate epileptiform discharges [16], suggesting that clonidine may open potassium channels. Further studies are needed to confirm this hypothesis.

In the present study, it has been shown that clonidine hyperpolarizes the membrane potential recorded in a zero-magnesium and elevated-potassium extracellular solution. This effect was diminished but not fully abolished in the presence of alpha-2 adrenergic receptor antagonist idazoxan. This suggests that clonidine hyperpolarizes the membrane potential not only via alpha-2 adrenergic receptor stimulation but also via direct influence on ionic channels. It may be speculated that clonidine inhibits (directly and indirectly via alpha-2 receptor stimulation) tonically active, sodium-permeable Ih channels, resulting in membrane potential hyperpolarization [17,18].

This study shows that clonidine simultaneously inhibits IEDs and hyperpolarizes the membrane potential. It may be speculated that membrane potential hyperpolarization contributes to the blockade of IEDs by clonidine. If this mechanism occurred, clonidine would have likely inhibited IEDs more strongly with greater hyperpolarization. However, this was not the case, as the tested drug inhibited IEDs to the same extent in the absence and presence of idazoxan when the hyperpolarization was larger and smaller, respectively (see Figure 1Ad,Bc). Thus, membrane potential hyperpolarization most likely did not contribute to the inhibition of IEDs exerted by the tested drug.

The concentration of clonidine used in our experiments (100 µM) is higher than the therapeutic concentration of this drug [19]. However, the concentration we used is similar to that used in other in vitro studies, making our results comparable with other publications [17,18].

IEDs occur in patients with epilepsy between seizures [1]. They may also be present in patients with ADHD and contribute to the symptoms of this disorder [1,4,5]. Clonidine is a drug used to treat ADHD. Other authors have shown that clonidine exerts beneficial effects in ADHD by stimulating alpha-2 adrenergic receptors [3]. This study shows that clonidine inhibits IEDs via a direct influence on ionic channels. It must be emphasized, however, that in vitro recordings of IEDs performed here do not fully reflect the complex pathophysiology of IEDs in ADHD in humans because brain slices were isolated from the surrounding brain structures and perfused with artificial proepileptic solution. Moreover, cortical slices were obtained from animals. For this reason, IEDs recorded in vitro are not fully comparable with IEDs in humans. Nevertheless, it may be purely hypothesized that the inhibition of IEDs by clonidine may, in some cases, contribute to beneficial effects of this drug in the treatment of ADHD, as IEDs may increase symptoms of this disorder [1,4,5].

This hypothesis, based on human studies [1,4,5], should be confirmed in an appropriate in vivo animal model of ADHD. There are, however, very few publications describing models in which interictal epileptiform discharges and ADHD-like symptoms coexist in laboratory animals. It has been reported that reductions in SNAP-25 (synaptosomal-associated protein of 25 kDa) levels were associated with moderate hyperactivity and interictal epileptiform discharges [20]. Importantly, it has been reported that interictal discharges induced in the prefrontal cortex of young rats cause attention deficits later in life [21], which can be improved by ACTH treatment. Further behavioral studies aimed at inhibiting IEDs in the models mentioned above should be conducted to confirm our in vitro findings in vivo.

## 4. Materials and Methods

The experimental procedures used in this study adhered to the Polish and international guidelines on the ethical use of animals (Directive 2010/63/EU, Polish legislation for the protection of animals used for scientific or educational purposes 2015). According to the Polish legislation, decapitation, which is carried out for the collection of brain tissue, does not require approval from a local ethical commission.

Male Wistar rats (3-week-old) were purchased from a local animal facility. Rats were bred at room temperature (12 h/12 h light/dark cycle) and fed a standard laboratory chow.

The animals were decapitated, and the brains were removed. Slices (300 µM) of the prefrontal cortex were prepared and incubated in exactly the same way as shown in our previous studies [7,22].

### 4.1. Recordings in Slices

Recordings were made from layer V pyramidal neurons located in the medial prefrontal cortex.

Interictal-like epileptiform discharges (IEDs) were recorded in the current-clamp configuration in zero-magnesium and elevated-potassium (5 mM) proepileptic extracellular solution, which contained (in mM): NaCl (130), KCl (5), glucose (10), NaHCO_3_ (25), NaH_2_PO_4_ (1.25), and CaCl_2_ (2); pH = 7.4.

Tonic NMDA currents were recorded in the voltage-clamp configuration in zero-magnesium extracellular solution, which contained (in mM): NaCl (130), KCl (2.5), glucose (10), NaHCO_3_ (25), NaH_2_PO_4_ (1.25), CaCl_2_ (2), and glycine (0.05); pH = 7.4. Magnesium ions were omitted and glycine was added to facilitate NMDA receptors. Moreover, this solution contained tetrodotoxin (TTX) 0.25 µM, DNQX 10 µM, and picrotoxin 50 µM to block synaptic transmission. NMDA 2 µM was applied to the bath. After a stable NMDA current was evoked, NMDA 2 µM and clonidine 100 µM were coapplied (see results).

Tonic AMPA currents were recorded in the voltage-clamp configuration in the extracellular solution, which contained (in mM): NaCl (130), KCl (2.5), glucose (10), NaHCO_3_ (25), NaH_2_PO_4_ (1.25), MgCl_2_ (1), and CaCl_2_ (2); pH = 7.4. This solution also contained TTX 0.25 µM, AP-5 25 µM, and picrotoxin 50 µM to block synaptic transmission. AMPA 1 µM was applied to the bath. After a stable AMPA current was evoked, AMPA 1 µM and clonidine 100 µM were coapplied (see results).

For all slice recordings, the intracellular solution in the patch pipette was composed of (in mM): potassium-gluconate (105), KCl (20), HEPES-Na^+^ (10), EGTA (0.1), MgATP (4), and GTP (0.5); pH = 7.4. Neurons were visualized in DIC optics. Slice recording techniques were the same as in our previous study [7,22]. Patch-pipettes had resistances between 4 and 5 MΩ. Recordings were obtained at 35 °C. Clonidine was applied to the bath.

### 4.2. Recordings in Dispersed Neurons

Sections of slices containing the medial prefrontal cortex were enzymatically and mechanically dispersed in exactly the same way as described in our previous study [7,22]. Recordings of fast-activating and fast-inactivating voltage-gated sodium currents were made from pyramidal neurons.

Sodium currents were recorded in an external solution with the following composition (in mM): NaCl (30), choline chloride (90), TEA-Cl (30), CaCl_2_ (2), MgCl_2_ (2), glucose (15), HEPES (10), CdCl_2_ (0.4), and LaCl_3_ (0.005); the pH was 7.4. Voltage-gated calcium currents were blocked by cadmium and lanthanum ions in the extracellular solution. Voltage-gated potassium currents were blocked by TEA-CL in the extracellular solution. Moreover, potassium ions were absent in the intracellular solution. The pipette (intracellular) solution contained the following (in mM): CsF (110), NaCl (7), EGTA (3), HEPES-Cl (10), MgCl_2_ (2), and Na_2_ATP (4); the pH was 7.4.

Recording techniques were exactly the same as in our previous publications [7,22]. The access resistance ranged from 5 to 7 MΩ. The currents were leak-subtracted. All recordings were performed at room temperature (21–22 °C). Clonidine was applied to the bath.

### 4.3. Statistical Analysis

Differences among more than two groups were evaluated using one-way repeated-measures ANOVA followed by Tukey’s post hoc test if the data passed the normality test. If the data did not pass the normality test, the nonparametric equivalent of one-way ANOVA for repeated measures (Friedman’s test) was used, followed by Dunn’s post hoc test. Depending on the results of the normality test, Student’s *t*-test or Wilcoxon matched-pairs test were used to evaluate differences between the two groups. The Shapiro–Wilk normality test was applied. Statistical analysis was made using GraphPad Prism 7 software.

## Data Availability

Data may be provided by the corresponding author on request.

## References

[1] Horvath A.A., Csernus E.A., Lality S., Kaminski R.M., Kamondi A. (2020). Inhibiting Epileptiform Activity in Cognitive Disorders: Possibilities for a Novel Therapeutic Approach. Front. Neurosci..

[2] Abdelnour E., Jansen M.O., Gold J.A. (2022). ADHD Diagnostic Trends: Increased Recognition or Overdiagnosis?. Mo. Med..

[3] Ji X.H., Ji J.Z., Zhang H., Li B.M. (2008). Stimulation of alpha2-adrenoceptors suppresses excitatory synaptic transmission in the medial prefrontal cortex of rat. Neuropsychopharmacology.

[4] Socanski D., Aurlien D., Herigstad A., Thomsen P.H., Larsen T.K. (2015). Attention deficit/hyperactivity disorder and interictal epileptiform discharges: It is safe to use methylphenidate?. Seizure.

[5] Han S.A., Yang E.J., Song M.K., Kim S.J. (2017). Effects of lamotrigine on attention-deficit hyperactivity disorder in pediatric epilepsy patients. Korean J. Pediatr..

[6] D’Antuono M., Köhling R., Ricalzone S., Gotman J., Biagini G., Avoli M. (2010). Antiepileptic drugs abolish ictal but not interictal epileptiform discharges in vitro. Epilepsia.

[7] Pasierski M., Kołba W., Szulczyk B. (2023). Guanfacine inhibits interictal epileptiform events and sodium currents in prefrontal cortex pyramidal neurons. Pharmacol. Rep..

[8] Tian K., Schmidt E.F., Lambe E.K. (2016). Serotonergic Suppression of Mouse Prefrontal Circuits Implicated in Task Attention. eNeuro.

[9] Isaev D., Ivanchick G., Khmyz V., Isaeva E., Savrasova A., Krishtal O., Holmes G.J., Maximyuk O. (2012). Surface charge impact in low magnesium model of seizure in rat hippocampus. J. Neurophysiol..

[10] Graebenitz S., Kedo O., Speckmann E.J., Gorji A., Panneck H., Volkmar H., Palomero-Gallagher N., Schleicher A., Zilles K., Pape H.C. (2011). Interictal-like network activity and receptor expression in the epileptic human lateral amygdala. Brain.

[11] Dupere J.R., Dale T.J., Starkey S.J., Xie X. (1999). The anticonvulsant BW534U87 depresses epileptiform activity in rat hippocampal slices by an adenosine-dependent mechanism and through inhibition of voltage-gated Na+ channels. Br. J. Pharmacol..

[12] Shao L.R., Wang G., Stafstrom C.E. (2018). The Glycolytic Metabolite, Fructose-1,6-bisphosphate, Blocks Epileptiform Bursts by Attenuating Voltage-Activated Calcium Currents in Hippocampal Slices. Front. Cell. Neurosci..

[13] Maurice N., Tkatch T., Meisler M., Sprunger L.K., Surmeier D.J. (2001). D1/D5 dopamine receptor activation differentially modulates rapidly inactivating and persistent sodium currents in prefrontal cortex pyramidal neurons. J. Neurosci..

[14] Wang W.-Z., Yuan W.-J., Pan Y.-X., Tang C.-S., Su D.-F. (2004). Interaction between clonidine and N-methyl-D-aspartate receptors in the caudal ventrolateral medulla of rats. Exp. Brain Res..

[15] Maruta T., Nemoto T., Satoh S., Kanai T., Yanagita T., Wada A., Tsuneyoshi I. (2011). Dexmedetomidine and clonidine inhibit the function of Na(v)1.7 independent of α(2)-adrenoceptor in adrenal chromaffin cells. J. Anesth..

[16] Wu X., Chen Z., Sun W., Wang G., Zhang L., Zhang Y., Zang K., Wang Y. (2019). Activation of Kir2.3 Channels by Tenidap Suppresses Epileptiform Burst Discharges in Cultured Hippocampal Neurons. CNS Neurol. Disord. Drug Targets.

[17] Parkis M.A., Berger A.J. (1997). Clonidine reduces hyperpolarization-activated inward current (Ih) in rat hypoglossal motoneurons. Brain Res..

[18] Grzelka K., Kurowski P., Gawlak M., Szulczyk P. (2017). Noradrenaline Modulates the Membrane Potential and Holding Current of Medial Prefrontal Cortex Pyramidal Neurons via beta(1)-Adrenergic Receptors and HCN Channels. Front. Cell. Neurosci..

[19] Keränen A., Nykänen S., Taskinen J. (1978). Pharmacokinetics and side-effects of clonidine. Eur. J. Clin. Pharmacol..

[20] Corradini I., Donzelli A., Antonucci F., Welzl H., Loos M., Martucci R., De Astis S., Pattini L., Inverardi F., Wolfer D. (2014). Epileptiform activity and cognitive deficits in SNAP-25(+/−) mice are normalized by antiepileptic drugs. Cereb. Cortex.

[21] Hernan A., Alexander A., Lenck-Santini P.P., Scott R.C., Holmes G.L. (2014). Attention deficit associated with early life interictal spikes in a rat model is improved with ACTH. PLoS ONE.

[22] Szulczyk B., Spyrka A. (2022). Menthol exerts TRPM8-independent antiepileptic effects in prefrontal cortex pyramidal neurons. Brain Res..

